# DPCPX, a selective adenosine A1 receptor antagonist, enhances the antidepressant-like effects of imipramine, escitalopram, and reboxetine in mice behavioral tests

**DOI:** 10.1007/s00210-018-1551-z

**Published:** 2018-08-09

**Authors:** Aleksandra Szopa, Ewa Poleszak, Karolina Bogatko, Elżbieta Wyska, Sylwia Wośko, Urszula Doboszewska, Katarzyna Świąder, Aleksandra Wlaź, Jarosław Dudka, Andrzej Wróbel, Piotr Wlaź, Anna Serefko

**Affiliations:** 1 Department of Applied Pharmacy, Medical University of Lublin, Chodźki 1, 20-093 Lublin, Poland; 2 Department of Pharmacokinetics and Physical Pharmacy, Collegium Medicum, Jagiellonian University, Medyczna 9, 30-688 Kraków, Poland; 3 Department of Animal Physiology, Institute of Biology and Biochemistry, Faculty of Biology and Biotechnology, Maria Curie-Skłodowska University, Akademicka 19, 20-033 Lublin, Poland; 4 Department of Pathophysiology, Medical University of Lublin, Jaczewskiego 8, 20-090 Lublin, Poland; 5 Chair and Department of Toxicology, Medical University of Lublin, Chodźki 8, 20-093 Lublin, Poland; 6 Second Department of Gynecology, Medical University of Lublin, Jaczewskiego 8, 20-090 Lublin, Poland

**Keywords:** DPCPX, Selective adenosine A1 receptor antagonist, Antidepressants, Forced swim test, Tail suspension test, Mice

## Abstract

The main goal of the present study was to evaluate the influence of the adenosine A1 receptor (A1R) antagonist — DPCPX — on depressive-like behavior in mice, as well as the effect of DPCPX on the activity of imipramine, escitalopram, and reboxetine, each at non-effective doses. The influence of DPCPX on behavior and its influence on the activity of selected antidepressants was evaluated in the forced swim test (FST) and the tail suspension test (TST) in mice. Locomotor activity was measured to verify and exclude false-positive data obtained in the FST and TST. Moreover, serum and brain concentrations of tested antidepressants were determined using HPLC. DPCPX, at doses of 2 and 4 mg/kg, exhibited antidepressant activity in the FST and TST, which was not related to changes in the spontaneous locomotor activity. Co-administration of DPCPX with imipramine, escitalopram, or reboxetine, each at non-active doses, significantly reduced the immobilization period in the FST and TST in mice, which was not due to the increase in locomotor activity. Both antagonists of 5-HT receptors (WAY 100635 and ritanserin) completely antagonized the effect elicited by DPCPX in the behavioral tests. Results of assessment of the nature of the interaction between DPCPX and test drugs show that in the case of DPCPX and imipramine or reboxetine, there were pharmacodynamic interactions, whereas the DPCPX-escitalopram interaction is at least partially pharmacokinetic in nature. Presented outcomes indicate that an inhibition of A1Rs and an increase of monoaminergic transduction in the CNS may offer a novel strategy for the development of antidepressant drugs.

## Introduction

It is known that adenosine is involved not only in the regulation of a wide range of behaviors, moods, and emotions (Boison 2008; Cunha et al. 2008; El Yacoubi et al. 2000; Ruby et al. 2010; Asatryan et al. 2011), but also cognitive processes (Kopf et al. 1999) and motor activity (Brockwell and Beninger 1996). Adenosine as a neuromodulator exerts its functions through the activation of four G-protein-coupled adenosine receptors (AR) — A1, A2A, A2B, and A3 (Fredholm et al. 2001, 2005a, b; Jacobson and Gao 2006; Boison 2008). Their roles and pharmacology have been analyzed in detail, with respect to control of transmitter release, modulation of neuronal excitability, and regulation of ion channel function (Fredholm et al. 1999, 2005a, b; Ferré et al. 2010).

Adenosine, adenosine analogs, and adenosine degradation inhibitors, which cause the non-selective AR activation, induce depressive-like behaviors in some animal models of depression (Kulkarni and Mehta 1985; Woodson et al. 1998). On the other hand, much data indicate that a non-selective pharmacological inhibition of adenosine receptors (e.g., administration of methylxanthines such as caffeine, theophylline) may reduce depressive-like behaviors in laboratory animals (Minor et al. 1994a, b; El Yacoubi et al. 2003; Minor and Hanff 2015; Szopa et al. 2016). However, Kaster et al. (2004, 2005a, b, 2007) presented the contrary data, which indicated that the non-selective activation of adenosine receptors decreased the immobilization period in the forced swim test (FST) and tail suspension test (TST) in mice.

Nowadays, due to the high stress accompanying everyday life, the number of patients suffering from mental illness increases annually. As a consequence, psychiatric disorders, including depression, have become one of the biggest problems worldwide (Wittchen et al. 2011; Olesen et al. 2012; WHO 2017). Despite the availability of a wide range of drugs for mental diseases with various mechanisms of action, therapeutic effects are still not optimal, and there is an urgent need to develop alternative therapeutic options. Recently, a particular attention has been given to a relationship between adenosine, adenosine receptors, and processes occurring in the brain under normal and disease conditions (Yamada et al. 2014), so they are perceived as important therapeutic targets (Chen et al. 2013; Sachdeva and Gupta 2013; Yamada et al. 2014; Vincenzi et al. 2016).

Considerable literature indicates that inhibition of adenosine neurotransmission may decrease the symptoms of mental illness, including depression, so it is of interest to determine the effect of A1R antagonist on the activity of commonly used antidepressants. The main goal of this study was to assess the effect of DPCPX — a selective A1-receptor antagonist — on animal behavior during short-term exposure to inescapable and uncontrollable stress. A further goal was to evaluate the influence of DPCPX on the activity of three common antidepressants representing different classes, imipramine — a tricyclic antidepressant (TCA), escitalopram — a selective serotonin reuptake inhibitor (SSRI), and reboxetine — a selective noradrenaline reuptake inhibitor (SNRI). Two behavioral tests widely used to determine antidepressant properties of drugs, the FST, and TST, were used. To verify and exclude false-positive/negative outcomes, spontaneous locomotor activity was measured. In order to elucidate the role of serotoninergic receptors 5-HT1A and 5-HT2 in the actions of tested substances, we used selective antagonists of these receptors — WAY 100635 and ritanserin, respectively. The effect of DPCPX on the level of antidepressants in murine serum and brain homogenates was estimated using high-performance liquid chromatography (HPLC).

## Materials and methods

### Animals

Adult male albino Swiss mice weighing 25–30 g form licensed breeder (Kołacz, Warsaw, Poland) were used for all experiments. The animals were housed in environmentally controlled rooms (temperature maintained at 21–25 °C and humidity 40–60%) in groups of ten in standard cages with unlimited access to water and food, with a 12 h light/dark cycle. The procedures began after at least a 1-week acclimatization period in the facility and were performed between 8 a.m. and 3 p.m. to minimize circadian influences. All procedures were conducted in accordance with the European Communities Council Directive and Polish legislation acts concerning animal experimentations. The procedures and protocols were approved by the First Local Ethics Committee at the Medical University of Lublin (license no 5/2015).

### Drug administration

DPCPX (8-cyclopentyl-1,3-dipropylxanthine, 1, 2, and 4 mg/kg, Sigma-Aldrich, Poznań, Poland) was suspended in a 1% aqueous solution of Tween 80 (POCH S.A., Gliwice, Poland). Imipramine hydrochloride (15 mg/kg, Sigma-Aldrich), reboxetine mesylate (2.5 mg/kg, Ascent Scientific, Cambridge, UK), escitalopram oxalate (2 mg/kg, Sigma-Aldrich), WAY 100635 (0.1 mg/kg, Sigma-Aldrich), and ritanserin (4 mg/kg, Sigma-Aldrich) were dissolved in 0.9% NaCl. All solutions were administrated intraperitoneally (*ip*) 60 min, whereas DPCPX suspension was injected *ip* 30 min prior to behavioral testing. The volume of all administrated solutions/suspension was 10 ml/kg. Time of drugs administration was chosen so that the behavioral tests were performed at the point of maximum effect of these substances. The doses and pretreatment schedules were selected on the basis of the literature and prior results (Poleszak 2007; Poleszak et al. 2005, 2011; Szewczyk et al. 2002, 2009; Szopa et al. 2016; Poleszak et al. 2016). In the studies in which the influence of DPCPX on the activity of common antidepressants was examined the non-active doses of DPCPX (1 mg/kg), and tested antidepressants were injected. In turn, in experiments with 5-HT receptor antagonists, an effective dose of DPCPX (2 mg/kg) was used to show whether the serotoninergic receptors 5-HT1A and 5-HT2 are involved in the operation of DPCPX antidepressant-like activity. Control groups received *ip* injections of saline.

### Forced swim test

The FST was carried out according to the method of Porsolt et al. (1977). Each mouse was placed individually for 6 min into a glass cylinder (height 25 cm, diameter 10 cm) containing 15 cm of water at 23–25 °C. After the first 2 min of the test, total duration of immobility was measured. A mouse was judged to be immobile when it ceased struggling and remained floating motionless and making only movements allowing to keep the head just above the surface of water. FST results are presented as the average duration of immobility time (seconds) ± standard error of the mean (SEM) for each experimental group.

### Tail suspension test

The TST was carried out according to the method of Steru et al. (1985). Each mouse was suspended individually for 6 min by the tail (2 cm from the end of the tail) using adhesive tape. After the first 2 min of the test, total duration of immobility was measured. A mouse was judged to be immobile when it ceased moving limbs and body, making only movements required to breathe. TST results are presented as the average duration of immobility time (seconds) ± SEM for each experimental group.

### Spontaneous locomotor activity

Spontaneous locomotor activity was measured using Opto-Varimex-4 Auto-Track (Columbus Instruments, Columbus, OH, USA) which consist of four cages made of Plexiglas with lids (43 × 43 × 32 cm). The cages were equipped with a set of four infrared emitters and four detectors, which monitor animal movements. Each mouse was placed individually for 6 min into a cage to determine the distance traveled by the animal between 2 and 6 min, which corresponds with the time interval analyzed in the FST and TST. Results obtained in the spontaneous locomotor activity test are presented as the average distance traveled by mice (cm) ± SEM for each experimental group.

### Determination of antidepressant drugs levels in serum and brains homogenates of mice

To obtain blood and brain for pharmacokinetic studies, mice were decapitated 60 min after administration of antidepressant drug with or without DPCPX. The blood was collected into Eppendorf tubes and allowed to cloth. Samples were then centrifuged for 10 min at 1000 rpm, and the centrifuged serum were collected into polyethylene tubes and frozen at − 25 °C. Brains, just after decapitation, were dissected from the skulls, rinsed with 0.9% NaCl, and frozen at − 25 °C.

Brain and serum concentrations of antidepressants were assayed by HPLC as described previously (Poleszak et al. 2016; Szopa et al. 2016).

Calibration curves constructed on the basis of the ratios of the peak heights of the tested drugs to the peak heights of the appropriate internal standards versus the known drug concentrations were linear in the tested concentration ranges. No interfering peaks were observed in the chromatograms. Assays were reproducible with low intra- and inter-day variation (a coefficient of variation less than 10%). The extraction efficiencies of the analyzed compounds and internal standards ranged from 66 to 97%. Concentrations of antidepressants were expressed in ng/ml of serum and ng/g of wet brain tissue.

### Statistical analysis

Statistical analysis was performed using one-way ANOVA with Dunnett’s post hoc, two-way ANOVA with Bonferroni’s post hoc test, or Student’s *t* test, depending on the study design. Results are considered statistically significant when the *p* values were ≤ 0.05.

## Results

### Forced swim test

#### DPCPX and antidepressants

**Fig. 1:**
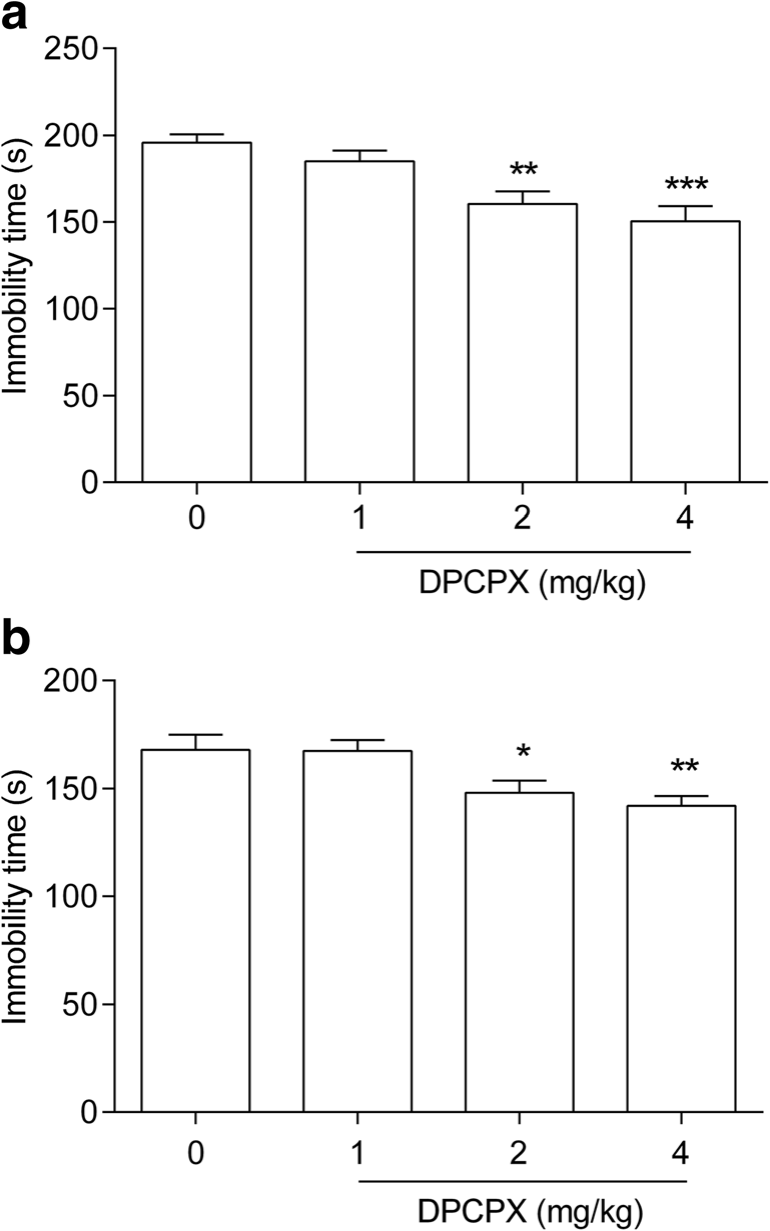
The antidepressant activity of DPCPX in the FST (**a**) and TST (**b**) in mice. DPCPX and saline were administered *ip* 30 min prior to the test. The data are presented as the means + SEM. Each experimental group consisted of ten animals. *p < 0.05, **p < 0.01, ***p < 0.001 vs control group (one-way ANOVA followed by Dunnett’s post hoc test)

DPCPX, at doses of 1, 2, and 4 mg/kg, was administered to ascertain the dose-effect relationship in the FST (Fig. 1a). DPCPX at doses of 2 and 4 mg/kg, but not 1 mg/kg, caused a significant shortening of the duration of immobility in the FST vs saline-treated group [one-way ANOVA F(3,33) = 9.196; **p < 0.01, ***p < 0.001, *p* > 0.05 respectively].

**Fig. 2:**
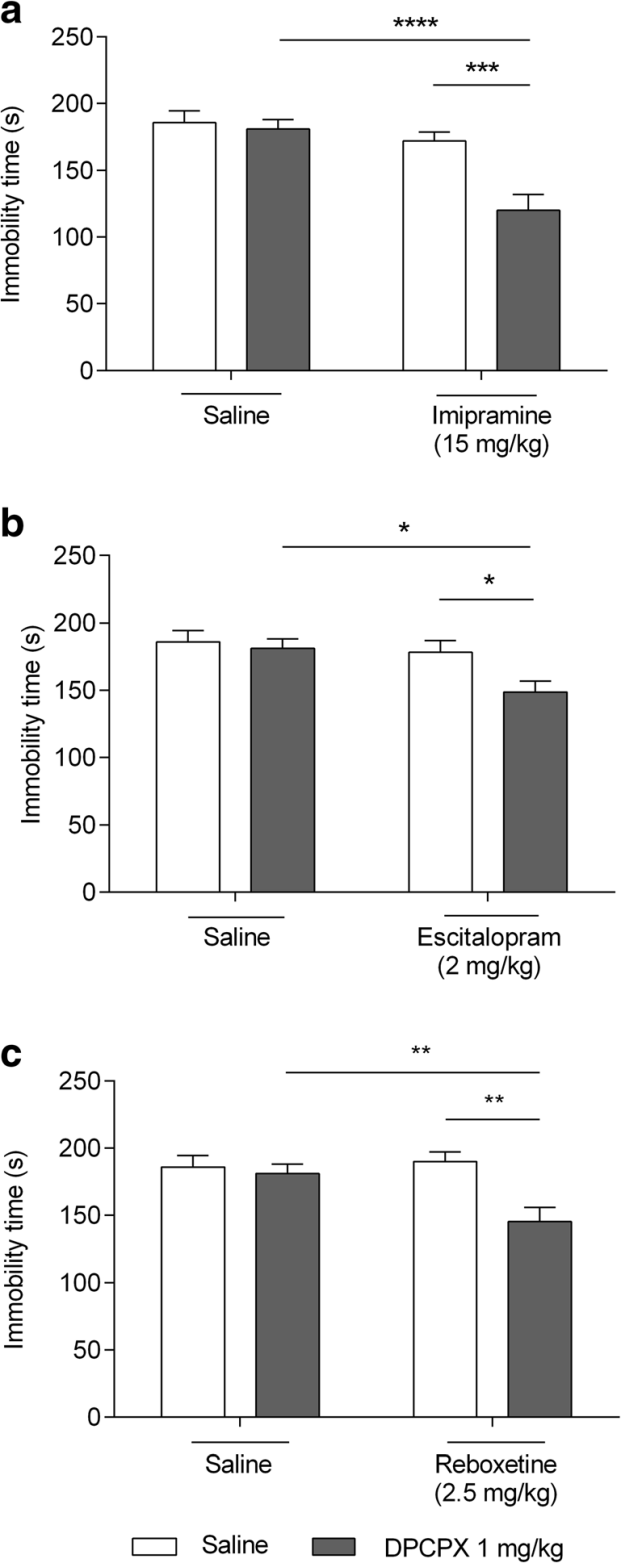
Effect of combined administration of DPCPX and antidepressants in the FST in mice. Antidepressants and saline were administered *ip* 60 min, whereas DPCPX *ip* 30 min prior to the test. The data are presented as the means + SEM. Each experimental group consisted of ten animals. **a**. ****p < 0.0001 vs DPCPX-treated group, ***p < 0.001 vs imipramine-treated group. **b**. *p < 0.05 vs DPCPX-treated group and escitalopram-treated group. **c**. **p < 0.01 vs DPCPX-treated group and reboxetine-treated group (two-way ANOVA followed by Bonferroni’s post hoc test)

Imipramine (15 mg/kg) did not statistically significant changes in the FST (*p* > 0.05). Significant immobility time reduction was noted when DPCPX and imipramine were co-administered in non-effective doses (1 and 15 mg/kg, respectively) (*p* < 0.0001 vs DPCPX-treated group, *p* < 0.001 vs imipramine-treated group) (Fig. 2a). A significant effect of imipramine [F(1,36) = 18.33, *p* = 0.0001], a significant effect of DPCPX [F(1,36) = 10.52, *p* = 0.0025], and a significant interaction between imipramine and DPCPX [F(1,36) = 7.27, *p* = 0.0106] were shown in the two-way ANOVA analysis.

Escitalopram (2 mg/kg) did not cause statistically significant changes in the FST (*p* > 0.05). Significant immobility time reduction was noted when DPCPX and escitalopram were co-administered in non-effective doses (1 and 2 mg/kg, respectively) (*p* < 0.05 vs DPCPX-treated group and escitalopram-treated group) (Fig. 2b). A significant effect of escitalopram [F(1,35) = 6.115, *p* = 0.0184], a significant effect of DPCPX [F(1,35) = 4.435, *p* = 0.0424], and no interaction between escitalopram and DPCPX were shown in the two-way ANOVA analysis.

Reboxetine (2.5 mg/kg) did not cause statistically significant changes in the FST (*p* > 0.05). Significant immobility time reduction was noted when DPCPX and reboxetine were co-administered in non-effective doses (1 and 2.5 mg/kg, respectively) (*p* < 0.01 vs DPCPX-treated group and reboxetine-treated group) (Fig. 2c). No effect of reboxetine [F(1,36) = 3.55, *p* = 0.0676], a significant effect of DPCPX [F(1,36) = 8636, *p* = 0.0057], and a significant interaction between reboxetine and DPCPX [F(1,36) = 5.617, *p* = 0.0233] were shown in the two-way ANOVA analysis.

#### 5-HT receptor antagonists and intrinsic effects of DPCPX

WAY 100635 influenced DPCPX antidepressant-like activity in the FST as demonstrated in Fig. 4a. DPCPX (2 mg/kg), but not WAY 100635 (0.1 mg/kg), produced a statistically significant change in animal behavior in the FST (*p* < 0.01 and *p* > 0.05, respectively). The antidepressant-like effect of DPCPX (2 mg/kg) was reduced by the injection of WAY 100635 at a dose of 0.1 mg/kg (*p* < 0.0001 vs DPCPX-treated group). A significant effect of WAY 100635 [F(1,26) = 15.91; *p* = 0.0005], no effect of DPCPX [F(1,26) = 3.748; *p* = 0.0638], and a significant interaction between WAY 100.635 and DPCPX [F(1,26) = 9.273; *p* = 0.0053] were shown in the two-way ANOVA analysis.

Ritanserin influenced DPCPX antidepressant-like activity in the FST as demonstrated in Fig. 4b. DPCPX (2 mg/kg), but not ritanserin (4 mg/kg), produced a statistically significant change in animal behavior in the FST (*p* < 0.01 and *p* > 0.05, respectively). Antidepressant-like effect of DPCPX (2 mg/kg) was reduced by the injection of ritanserin at a dose of 4 mg/kg (*p* < 0.001 vs DPCPX-treated group). A significant effect of ritanserin [F(1,25) = 16.41; *p* = 0.0004], no effect of DPCPX [F(1,25) = 3.094; *p* = 0.0908], and a significant interaction between ritanserin and DPCPX [F(1,25) = 8.298; *p* = 0.0080] were shown in the two-way ANOVA analysis.

### Tail suspension test

#### DPCPX and antidepressants

DPCPX, at doses of 1, 2, and 4 mg/kg, was administered to ascertain the dose-effect relationship in the TST (Fig. 1b). DPCPX at doses of 2 and 4 mg/kg, but not 1 mg/kg, caused a significant shortening of the duration of the animals’ immobility in the FST vs saline-treated group [one way ANOVA F(3,36) = 6.239, *p < 0.05, **p < 0.01, *p* > 0.05 respectively].

**Fig. 3:**
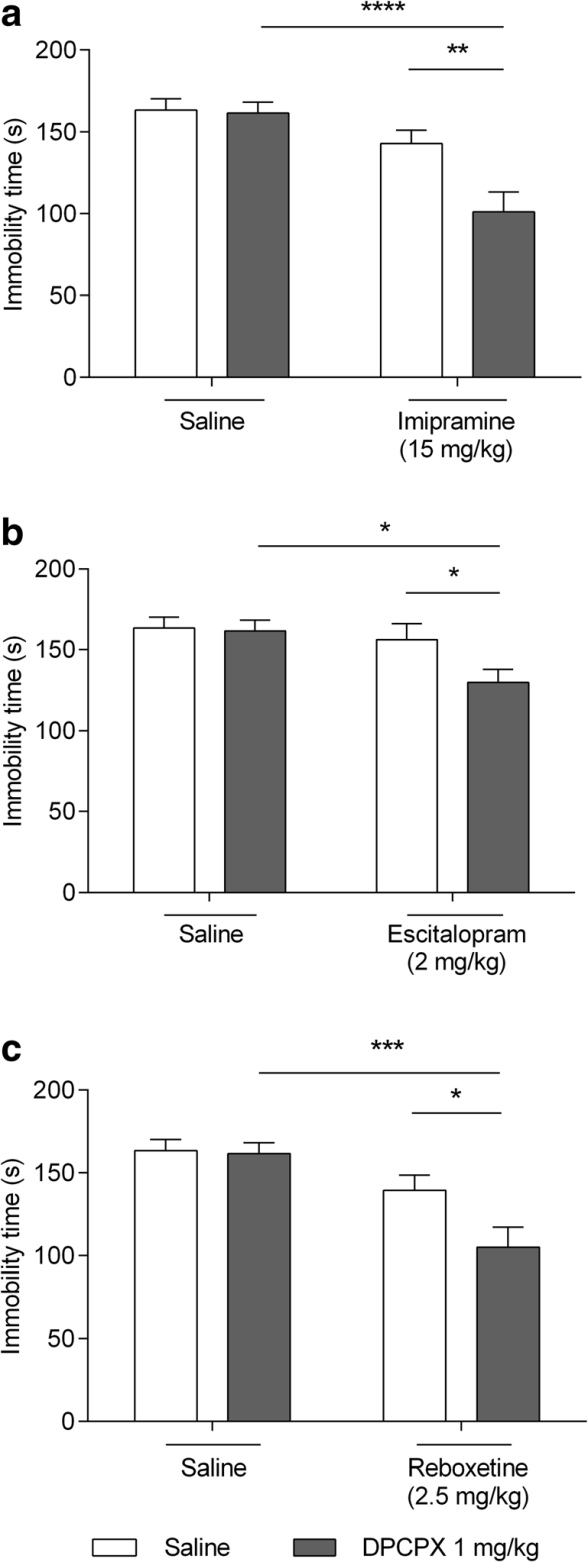
Effect of combined administration of DPCPX and antidepressants in the TST in mice. Antidepressants and saline were administered *ip* 60 min, whereas DPCPX *ip* 30 min prior the test. The data are presented as the means + SEM. Each experimental group consisted of ten animals. **a**. ****p < 0.0001 vs DPCPX-treated group, **p < 0.01 vs imipramine-treated group. **b**. *p < 0.05 vs DPCPX-treated group and escitalopram-treated group. **c**. ***p < 0.01 vs DPCPX-treated group, *p < 0.05 vs reboxetine-treated group (two-way ANOVA followed by Bonferroni’s post hoc test)

Imipramine (15 mg/kg) did not cause statistically significant changes in the TST (*p* > 0.05). Significant immobility time reduction was noted when DPCPX and imipramine were co-administered in non-effective doses (1 and 15 mg/kg, respectively) (*p* < 0.0001 vs DPCPX-treated group, *p* < 0.01 vs imipramine-treated group) (Fig. 3a). A significant effect of imipramine [F(1,36) = 21.39, *p* < 0.001], a significant effect of DPCPX [F(1,36) = 6.255, *p* = 0.0171], and a significant interaction between imipramine and DPCPX [F(1,36) = 5.217, *p* = 0.0284] were shown in the two-way ANOVA analysis.

Escitalopram (2 mg/kg) did not cause statistically significant changes in the TST (*p* > 0.05). Significant immobility time reduction was noted when DPCPX and escitalopram were co-administered in non-effective doses (1 and 2 mg/kg, respectively) (*p* < 0.05 vs DPCPX-treated group and escitalopram-treated group) (Fig. 3b). A significant effect of escitalopram [F(1,36) = 6.006, *p* = 0.0192], no effect of DPCPX [F(1,36) = 3.179, *p* = 0.0830], and no interaction between escitalopram and DPCPX [F(1,36) = 2.383, *p* = 0.1314] were shown in the two-way ANOVA analysis.

Reboxetine (2.5 mg/kg) did not cause statistically significant changes in the TST (*p* > 0.05). Significant immobility time reduction was noted when DPCPX and reboxetine were co-administered in non-effective doses (1 and 2.5 mg/kg, respectively) (*p* < 0.001 vs DPCPX-treated group, *p* < 0.01 vs reboxetine-treated group) (Fig. 3c). A significant effect of reboxetine [F(1,36) = 20.08, *p* < 0,001], no effect of DPCPX [F(1,36) = 4.072, *p* = 0.0511], and no interaction between reboxetine and DPCPX [F(1,36) = 3.262, *p* = 0.0793] were shown in the two-way ANOVA analysis.

#### 5-HT receptor antagonists and intrinsic effects of DPCPX

**Fig. 4:**
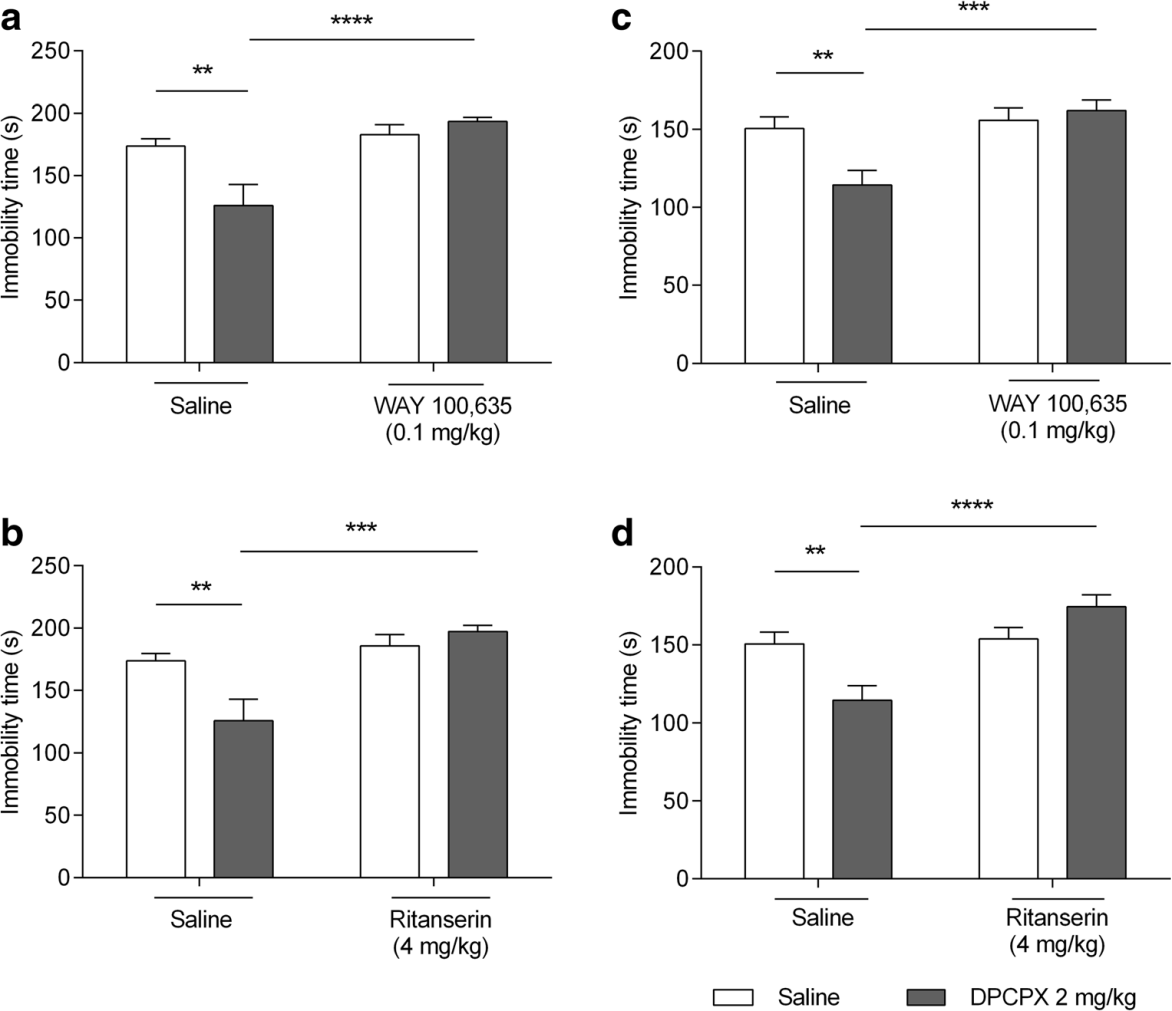
Effect of combined administration of DPCPX and selective antagonists of serotonin receptors 5-HT_1A_ and 5-HT_2_ in the FST (**a**, **b**) and TST (**c**, **d**) in mice. WAY 100635, ritanserin, and saline were administered *ip* 60 min, whereas DPCPX *ip* 30 min prior to the test. The data are presented as the means + SEM. Each experimental group consisted of ten animals. **p < 0.01 vs control group, ***p < 0.001, ****p < 0.0001 vs DPCPX-treated group (two-way ANOVA followed by Bonferroni’s post hoc test)

WAY 100635 influenced DPCPX antidepressant-like activity in the TST as demonstrated in Fig. 4c. DPCPX (2 mg/kg), but not WAY 100635 (0.1 mg/kg), produced a statistically significant change in animal behavior in the FST (*p* < 0.01 and *p* > 0.05, respectively). The antidepressant-like effect of DPCPX (2 mg/kg) was reduced by the injection of WAY 100635 at a dose of 0.1 mg/kg (*p* < 0.001 vs DPCPX-treated group). A significant effect of WAY 100635 [F(1,28) = 10.99; *p* = 0.0025], no effect of DPCPX [F(1,28) = 3.567; *p* = 0.0693], and a significant interaction between WAY 100635 and DPCPX [F(1,28) = 7.058; *p* = 0.0129] were shown in the two-way ANOVA analysis.

Ritanserin influenced DPCPX antidepressant-like activity in the TST as demonstrated in Fig. 4d. DPCPX (2 mg/kg), but not ritanserin (4 mg/kg), produced a statistically significant change in animal behavior in the TST (*p* < 0.01 and *p* > 0.05, respectively). The antidepressant-like effect of DPCPX (2 mg/kg) was reduced by the injection of ritanserin at a dose of 4 mg/kg (*p* < 0.0001 vs DPCPX-treated group). A significant effect of ritanserin [F(1,28) = 15.55; *p* = 0.0005], no effect of DPCPX [F(1,28) = 0.9525; *p* = 0.3374], and a significant interaction between ritanserin and DPCPX [F(1,28) = 12.62; *p* = 0.0014] were shown in the two-way ANOVA analysis.

### Spontaneous locomotor activity

The effect of DPCPX (1, 2, and 4 mg/kg) and combined administration of DPCPX and the tested antidepressants on spontaneous locomotor activity in mice is shown in Tables 1 and 2. DPCPX (1, 2, and 4 mg/kg), imipramine (15 mg/kg), escitalopram (2 mg/kg), reboxetine (2.5 mg/kg), and WAY 100635 (0.1 mg/kg) given alone or in combination had no statistically significant effects on locomotor activity in mice. A single injection of ritanserin (4 mg/kg) and combined administration of DPCPX with ritanserin significantly (*p* < 0.001) shortened the locomotor activity in mice.

**Table 1:** Effect of DPCPX on locomotor activity in mice

Treatment (mg/kg)	Distance traveled (cm)
Saline (control group)	638.4 ± 85.12
DPCPX 1	589.4 ± 60.34
DPCPX 2 DPCPX 4	425.6 ± 60.13 487.6 ± 78.95

DPCPX and saline were administered *ip* 30 min prior to the test. Distance traveled was recorded between the 2nd and the 6th min of the test. The data are presented as the means ± SEM. Each experimental group consisted of eight animals. The results were considered statistically significant if *p* < 0.05 (one-way ANOVA followed by Dunnett’s post hoc test)

**Table 2:** Effect of treatments on spontaneous locomotor activity in mice

	Treatment (mg/kg)	Distance traveled (cm)
(A)	Saline + saline	490.6 ± 50.75
	DPCPX 1 + saline	487.9 ± 72.85
	Imipramine 15 + saline	547.1 ± 64.74
	DPCPX 1 + imipramine 15	476.4 ± 80.00
	Escitalopram 2 + saline	542.0 ± 58.06
	DPCPX 1 + escitalopram 2	543.5 ± 63.09
	Reboxetine 2.5 + saline	456.6 ± 68.06
	DPCPX 1 + reboxetine 2.5	485.6 ± 47.43
(B)	Saline + saline	538.9 ± 72.09
	DPCPX 2 + saline	516.0 ± 53.41
	WAY 0.1 + saline	494.6 ± 41.80
	DPCPX 2 + WAY 0.1	513.4 ± 70.12
	Ritanserin 4 + saline	92.43 ± 36.67***
	DPCPX 2 + ritanserin 4	126.1 ± 43.53***

Antidepressants, WAY, ritanserin, and saline were administered *ip* 60 min, whereas DPCPX *ip* 30 min prior to the test. Distance traveled was recorded between the 2nd and the 6th min of the test. Each experimental group consisted of eight animals. Data are presented as the means ± SEM. ***p < 0.001 vs DPCPX-treated and control group (two-way ANOVA followed by Bonferoni’s post hoc test)

The two-way ANOVA demonstrated: (A) no effect of imipramine [F(1,28) = 0.1096, *p* = 0.7431], no effect of DPCPX [F(1,28) = 0.2924, *p* = 0.5930], and no interaction [F(1,28) = 0.2503, *p* = 0.6208]. (B) no effect of escitalopram [F(1,28) = 0.7513; *p* = 0.3934], no effect of DPCPX [F(1,28) = 0.0001025, *p* = 0.9920], and no interaction [F(1,28) = 0.001185, *p* = 0.9728]. (C) no effect of reboxetine [F(1,28) = 0.08898, *p* = 0.7677], no effect of DPCPX [F(1,28) = 0.04666, *p* = 0.8305], and no interaction [F(1,28) = 0.06826, *p* = 0.7958]. (D) no effect of WAY 100635 [F(1,28) = 0.1493, *p* = 0.7021], no effect of DPCPX [F(1,28) = 0.001156; *p* = 0.9731], and no interaction [F(1,28) = 0.1178, *p* = 0.7340]. (E) a significant effect of ritanserin [F(1,27) = 59.45, *p* < 0,001], no effect of DPCPX [F(1,27) = 0.009954, *p* = 0.9213], and no interaction [F(1,27) = 0.2720, *p* = 0.6062].

### Pharmacokinetic studies

The effect of DPCPX on serum and brain concentrations of antidepressants in mice is shown in Table 3. In the case of combined administration of DPCPX with imipramine and reboxetine, no significant changes in drug concentration were observed in murine serum and brain homogenates. DPCPX increased the concentration of escitalopram (*t* test *p* < 0.01) in brain tissue without significant changes in serum (*t* test *p* > 0.05).

**Table 3:** Effect of DPCPX on the concentration of antidepressants in mouse serum and brain

Treatment (mg/kg)		Antidepressants concentration in serum (ng/ml)		Antidepressants concentration in brain (ng/g)	
(A)	Imipramine 15 + saline	328.7 ± 80.42		5522 ± 943.7	
	(Metabolite — desipramine)	(48.69 ± 7.070)		(170.0 ± 50.94)	
	Imipramine 15 + DPCPX 1	225.1 ± 36.19	*p* = 0.2556	5735 ± 894.2	*p* = 0.8717
	(Metabolite — desipramine)	(36.29 ± 3.307)	*p* = 0.1444	(212.0 ± 22.35)	*p* = 0.7695
(B)	Escitalopram 2 + saline	57.51 ± 7.092		595.1 ± 65.31	
	Escitalopram 2 + DPCPX 1	66.27 ± 6.603	*p* = 0.3783	816.4 ± 38.35**	*p* = 0.0091
(C)	Reboxetine 2.5 + saline	99.60 ± 11.67		208.5 ± 28.37	
	Reboxetine 2.5 + DPCPX 1	110.0 ± 15.33	*p* = 0.5975	193.4 ± 18.91	*p* = 0.6642

Antidepressants were administered *ip* 60 min, whereas DPCPX *ip* 30 min prior to decapitation. Each experimental group consisted of ten animals. Results are presented as mean values ± SEM. **p < 0.01 vs respective control group (Student’s *t* test)

## Discussion

### DPCPX and antidepressant drug activity in the FST and TST

Behavioral despair and learned helplessness are typical symptoms of depressive disorders, and adenosine systems may be involved. Outcomes obtained by Woodson et al. suggested that an essential constituent in behavior induced by stress are adenosine and its receptor activation (Woodson et al. 1998). Moreover, Minor et al. indicated that depression-like effects in rodents are induced by administration either of a non-selective AR agonist (NECA) or a highly selective A1R agonist (R-PIA) (Minor et al. 1994b). Antidepressant-like activity of the non-selective ARs antagonist caffeine has been demonstrated in the FST and TST, and such effects were comparable with that of TCAs (Enríquez-Castillo et al. 2008; Vieira et al. 2008; Gan 2009; Szopa et al. 2016).

In the present study, the antidepressant-like effect of DPCPX in the FST and TST in mice has been shown. Doses of 2 and 4 mg/kg produced a significant reduction in the immobility time of animals in carried out behavioral tests, whereas the lowest dose of DPCPX − 1 mg/kg — did not exhibit antidepressant-like activity. The highest density of A1Rs is found in the brain, especially in the hippocampus, cortex, and striatum (Ochiishi et al. 1999; Hoyer et al. 2002; Hannon and Hoyer 2008; Wei et al. 2011). A1Rs stimulation leads to an inhibition of several neurotransmitter release and a reduction in postsynaptic excitability (Dunwiddie and Masino 2001). Inversely, an inhibition of these receptors causes a stimulation of neurotransmitter release (e.g., ACh, 5-HT, NA, DA) (Nestler et al. 2002). Shortening of the immobility duration in the FST and TST after administration of the selective A1R antagonist DPCPX, which has a xanthine structure, is probably the result of increased serotonergic, noradrenergic, and dopaminergic transduction (Müller and Scior 1993; Ferré et al. 1996; Müller and Stein 1996; Fredholm et al. 2005b; Górska and Gołembiowska 2015). These outcomes seem to be in disagreement to data that enhancement of adenosine is a possible treatment strategy for depression (Kaster et al. 2004).

The present findings demonstrate that DPCPX affects the action of imipramine, escitalopram, and reboxetine, and these are novel observations. Simultaneous administration of DPCPX and these agents at non-active doses resulted in a statistically significant reduction in the immobility times in either the FST or TST. Also, Herbet et al. have shown recently that co-administration of other selective A1R antagonist — CPT (8-cyclopentyl-1,3-dimethylxanthine, 3 mg/kg) — with imipramine at a non-active doses resulted in a statistically significant reduction in the immobility times during the behavioral test using short-term exposure to inescapable and/or uncontrollable stress (Herbet et al. 2018). Synergism of antidepressant actions was also observed using concomitant administration of TCAs, SSRIs, and SNRIs and the non-selective AR antagonist — caffeine — at ineffective doses in the mice FST (Szopa et al. 2016). The excitation of AR by adenosine, adenosine analogs, and selective AR agonists modulates serotonergic and dopaminergic neurotransmission (Regenold and Illes 1990; Okada et al. 2001; Yamato et al. 2002), and consequently decreases levels of ACh, 5-HT, and DA in the CNS (Okada et al. 2002). Furthermore, these substances suppress medicinal effects of commonly used antidepressants (Barcellos et al. 1998). Drugs which increase levels of monoamines such as 5-HT, NA, and DA in the CNS play a vital role in antidepressant therapy. DPCPX selectively influences A1Rs, which are presented on serotonergic neurons in the locus coeruleus (Regenold and Illes 1990) and the dorsal raphe nucleus (Mössner et al. 2000). The non-selective and selective blockage of A1Rs inhibits effects of endogenous adenosine and cause the opposite effect with regard to the NA and 5-HT transduction (Müller and Scior 1993; Müller and Stein 1996; Fredholm et al. 2005a, b). Preclinical and clinical studies show that antidepressant treatment affects ARs and modify behavioral responses. For example, TCAs are capable of attaching to ARs causing a reduction of extracellular adenosine level in the CNS synapses (Barcellos et al. 1998). All tested antidepressant agents modulate monoaminergic transmission: imipramine non-selectively inhibits neuronal NA and 5-HT reuptake (Sulser et al. 1962), escitalopram is the selective 5-HT reuptake inhibitor (Bræstrup and Sanchez 2004), while reboxetine selectively blocks neuronal reuptake of NA in the CNS (Hajós et al. 2004). The above effects may explain synergy observed between DPCPX and imipramine, escitalopram, and reboxetine in the present study.

### 5-HT receptor antagonists and intrinsic effects of DPCPX

The serotonin 5-HT1A receptor is an autoreceptor bestead on serotonergic neurons in the raphe nuclei (Sprouse and Aghajanian 1988) and is also found as a postsynaptic receptor localized in the hippocampus and amygdala (Chalmers and Watson 1991). In turn, the 5-HT2 receptor is located mainly on the postsynaptic serotonergic neurons in forebrain (López-Giménez et al. 1997). A1Rs are found in close proximity of 5-HT1 and 5-HT2A receptors. A high level of A1R expression was found in the cerebral cortex, hippocampus, cerebellum, striatum, and in the thalamic nuclei (Hoyer et al. 2002; Hannon and Hoyer 2008). This arrangement of receptors may indicate their mutual interaction. Due to the high probability that DPCPX affects serotonergic transmission based on common localization of receptors, an attempt was made to elucidate the involvement of serotonin 5-HT1A and 5-HT2A/2C receptor in its action. In our study, we determined whether pharmacological antagonism of 5-HT1A or 5-HT2 receptors (WAY 100635 and ritanserin, respectively) would modulate DPCPX activity in the FST and TST. Results show that WAY 100635 (0.1 mg/kg) and ritanserin (4 mg/kg) completely antagonized the effect of DPCPX (2 mg/kg) in both tests. Antidepressant-like activity of DPCPX in the FST and TST appears to be dependent, at least in part, on the serotonergic transmission via 5-HT1A and 5-HT2A/C. Supporting our proposal, results obtained by Detke et al. (1995), Redrobe et al. (1996), Redrobe and Bourin (1997), and Abert and Lemonde (2004) which demonstrated that 5-HT1A and 5-HT2A/C receptors participate in SSRIs antidepressant-like effect in rodent screening tests.

### Spontaneous locomotor activity

Since it is widely known that the antidepressant-like effect in the FST and TST may be evoked by the substances which induce hyperactivity, the influence of DPCPX and its combination with antidepressants on the spontaneous locomotor activity was evaluated. Shortening of the duration of immobility observed in presented studies was not associated with the increase of spontaneous locomotor activity. This is consistent with the results in Suzuki et al., which demonstrated that A1-selective antagonists did not increase spontaneous locomotion (Suzuki et al. 1993). Only ritanserin combined with DPCPX inhibited spontaneous locomotor activity. In other studies, similar effects on locomotor activity were observed after administration of ritanserin and compounds with potential antidepressant-like activity (Szewczyk et al. 2009; Poleszak et al. 2016).

### Pharmacokinetic studies

Pharmacokinetic investigation in the present study allowed us to assess concentrations of imipramine, escitalopram, and reboxetine in mice blood and brain after their combined administration with DPCPX and was aimed at determining drug-drug interactions involving changes in drug disposition. The present work is the first study in which such an attempt was made. There is no information about DPCPX pharmacokinetics in rodents or humans. Because DPCPX is a xanthine derivative (Müller and Jacobson 2011), it is likely that its metabolism is similar to that of other xanthines, e.g., caffeine and theophylline. The role of cytochromes P450 (CYPs) in the oxidative biotransformation of most drugs is well documented (Caccia 1998; Nelson et al. 2004; Guengerich 2008; Zanger et al. 2008). CYP1A2, the main isoenzyme responsible for the metabolism of xanthines, including caffeine, is also involved in metabolism of commonly used antidepressant drugs (Caccia 1998). Based on HPLC analysis of murine blood and brain tissue, we found that DPCPX does not statistically significantly affect the concentrations of imipramine, its active metabolite desipramine, and reboxetine. In the case of DPCPX and escitalopram, a significant increase in levels of antidepressant drug in the brain but no changes in the serum were noted. These outcomes suggest that DPCPX-impramine and DPCPX-reboxetine interaction are probably due to changes at the cellular level, so are pharmacodynamic in nature. Augmentation of escitalopram levels in the brain may be due to the facilitated transport of this drug through the blood-brain barrier (Pardridge 2005, 2007) after concomitant injection with DPCPX. The increase in antidepressant-like activity of escitalopram observed in the behavioral tests may be partly the result of pharmacokinetic interaction between DPCPX and escitalopram.

## Conclusions

In summary, we demonstrated that DPCPX produced an antidepressant-like effect. Furthermore, DPCPX significantly augmented the antidepressant-like potential of a TCA, SSRI, and SNRI without affecting spontaneous locomotor activity. The simultaneous blockage of A1Rs and increase of monoamine transduction in the CNS may offer an alternative target in the development of new options for the pharmacological treatment in patients with depression. In the near future, we are planning to perform studies using chronic concomitant administration of A1Rs antagonists and commonly used antidepressants.
